# Extra-Virgin Olive Oil Extracted Using Pulsed Electric Field Technology: Cultivar Impact on Oil Yield and Quality

**DOI:** 10.3389/fnut.2019.00134

**Published:** 2019-09-04

**Authors:** Gianluca Veneziani, Sonia Esposto, Agnese Taticchi, Roberto Selvaggini, Beatrice Sordini, Antonietta Lorefice, Luigi Daidone, Mauro Pagano, Roberto Tomasone, Maurizio Servili

**Affiliations:** ^1^Department of Agricultural, Food and Environmental Sciences, University of Perugia, Perugia, Italy; ^2^Council for Agricultural Research and Economics Research Centre for Engineering and Agro-Food Processing, Monterotondo, Italy

**Keywords:** extraction process, pulsed electric field, cultivar, oil yield, phenols, quality

## Abstract

The main operators of the olive oil sector are continuously involved in the development of the olive oil mechanical extraction process with the common aim of increasing both the quality and the oil extraction yield coupled with the potential enhancement of the working efficiency of the olive mill. The pulsed electric field (PEF) is a recently studied technological innovation for the improvement of olive oil extraction technology. The impact of the PEF on the diffusion of oil and microconstituents, determined by the disruption effects on olive cell tissues carried out by the non-thermal method, was evaluated. A PEF can increase the permeability and breaking of the cell membranes with a consequent positive result on oil extractability and quality, mainly related to the compounds involved in the health and sensory properties of extra virgin olive oil. The PEF was tested on three Italian olive cultivars (Carolea, Coratina, and Ottobratica). The results showed a positive impact of the new technology on the oil yield, with an increase ranging from 2.3 to 6%, and on the concentration of hydrophilic phenols, with an increase ranging from 3.2 to 14.3%, with respect to the control tests. The data of the main compounds related to the health and sensory notes also showed high variability as a consequence of the genetic origins of the olive cultivars.

## Introduction

The technological evolution of the olive oil mechanical extraction process in recent decades has been mainly based on the improvement of extra-virgin olive oil (EVOO) quality, which is strictly connected with compounds characterized by their health and sensory properties (phenolic and volatile compounds). The activities concerning the control of the main technological parameters (time, temperature, and oxygen) and the critical steps of the crushing and malaxation phases (1–4) benefitted from the introduction of heat exchangers that could easily and rapidly regulate the temperature of the crushed olive paste in relation to the climatic conditions of the harvesting period and the specific needs of the olive mill to achieve a better quality product (5–9).

In contrast, the recent technologies applied to the extraction system, such as microwaves, ultrasounds, and pulsed electric fields (PEFs), are mainly focused on increasing oil extractability and improving plant working efficiency with little attention on their impacts on the legal, commercial, and quality parameters of EVOO (10–15). All of these recent technologies are based on the degradation of the olive fruit cells through thermal and non-thermal treatments that enable pore formation, membrane permeability alterations, water influx, swelling, and deflation with an overall consequence of rupturing the cell walls and membranes. Cell lysis results in an abundant release of micro- and macro-intracellular components into the water phase that leads to an increase in free olive droplets characterized by different qualitative and quantitative chemical compositions due to the destructive effects on the olive tissues altering the solubilization phenomena and improving the mass transfer rate (16–18). The use of PEFs for the improvement of the quality characteristics of different foods and beverages is mainly linked to the enhancement of quality attributes, such as color, texture, flavor, phenolic compounds, carotenoids, and vitamins, and bioactive compound extractability, and thus, PEFs have been investigated in recent years (19–25). The application of the PEF system to the virgin olive oil extraction process, and to the valorization opportunities of by-product (26, 27), is still very limited, and there are only a few preliminary studies concerning its effects on oil yield and quality. Both Abenoza et al. (10) and Puértolas and Martínez de Marañón (14) analyzed the impact of the new technology on the oil extractability and chemical and sensory parameters by processing Arbequina and Arroniz olives and carrying out PEF treatment on the olive pastes before and after the malaxation phase, respectively. The first author used a laboratory-scale extraction system equipped with a PEF system set up at an electric field strength of 1 and 2 kV cm^−1^ and a frequency of 125 Hz. The treatment of the crushed paste did not result in any significant increase in oil yield but was able to guarantee the same extractability at a reduced malaxation temperature with a consequent positive impact on the EVOO sensory notes. The other study (14) investigated the activity of PEF of 2 kV cm^−1^ applied to the olive paste at a frequency of 25 Hz before the horizontal centrifuge using an industrial oil extraction plant. The non-thermal treatment resulted in an increase in the oil extraction yield and an improvement in the VOO quality related to the enhancement of the polyphenol, phytosterol and tocopherol contents. However, both studies highlighted the need for further research to evaluate the influences of external factors, such as the cultivar, maturity index, temperature, and other process parameters, on the performance of PEF applied to the olive oil mechanical extraction system.

Based on the information from these recent studies, the cultivar impact on the yield and quality of EVOOs using a different PEF system, characterized by a different set up, was investigated. This study reports detailed data of the trials carried out for processing the olives belonging to different Italian cultivars with particular attention to the olive cell destruction process and the subsequent release of larger amounts of oil and the main components connected to the sensory and quality parameters of the olive oils, such as the contents of hydrophilic and lipophilic phenols and volatile compounds.

## Materials and Methods

### EVOO Mechanical Extraction Process

**Figure 1:**
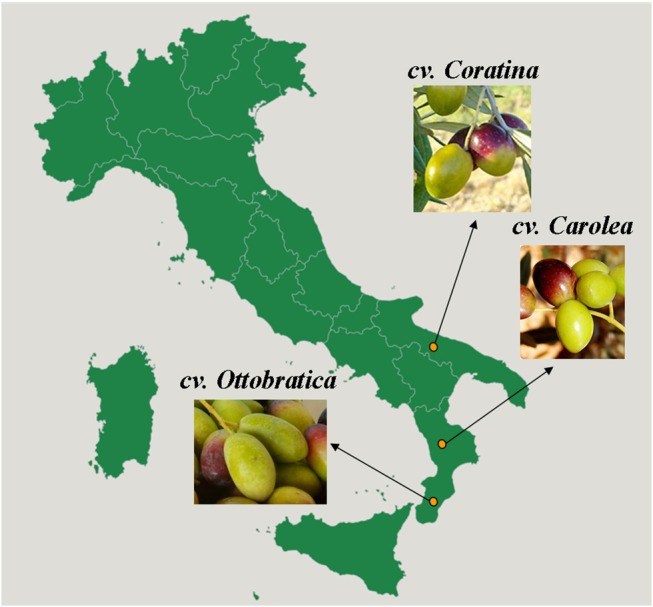
Geographical origin of three different Italian olive cultivars.

Control and PEF treated EVOO samples were extracted from the olives of the Carolea, Ottobratica, and Coratina cultivars (Figure 1). The olive batches of the Carolea (fruit weight: medium-high, high; stone size: large; fruit-flesh/pit ratio: medium, medium-high; oil content: medium, medium-high; tree-harvest time: medium, medium-late) and Ottobratica (fruit weight: medium-low, low; stone size: small; oil content: medium; tree-harvest time: early) cultivars were harvested in October 2017 in the Calabria region, whereas the olives of Coratina (fruit weight: medium, medium-high; stone size: large; fruit-flesh/pit ratio: low; oil content: high; tree-harvest time: medium-late) belonging to the Apulia region were purchased in the area of Bari during the first week of November 2017 (28). All olives were harvested at a medium-low maturity index ranging from 0.8 to 1.5 (29). The control and PEF EVOO samples were obtained in triplicate using an industrial plant TEM 200 system (Toscana Enologica Mori, Tavarnelle Val di Pesa, Florence, Italy) consisting of a hammer mill, a malaxer with a gas controller system and a working capacity of 200 kg of olives and a two-phase decanter; additionally a vertical centrifuge [UVPX 305 AGT 14 (Alfa Laval S.p.A., Tavarnelle Val di Pesa, Florence, Italy)] was used to separate the olive oil from the residual water phase. Single trials of 180 kg of olives each were carried out using a heat exchanger to determine the rapid thermal conditioning of olive pastes at 25°C ± 0.5 after the crushing phase. The oil extraction system was equipped with an oliveCEPT Model 6.2 (Arcaroma Pure AB, Lund, Sweden), which is a PEF system based on closed environment PEF treatment (CEPT) technology and positioned after the malaxation phase. The PEF was set up at an electric field strength of 1.7 kV cm^−1^ and a specific energy of 17 kJ kg^−1^.

### EVOO Analyses

During the experimental study, the main quality parameters potentially influenced by the introduction of the technological innovation of the olive oil mechanical extraction process were evaluated without analyzing other characteristics of EVOO rarely modified by olive oil mechanical extraction process such as fatty acid composition, sterols, and waxes (10, 14, 15).

### Chemicals

Phenolic alcohols such as hydroxytyrosol (3,4-DHPEA) and tyrosol (*p*-HPEA) were supplied by Cabru s.a.s. (Arcore, Milan, Italy) and Fluka (Milan, Italy), respectively. Lignans [(+)-1-acetoxypinoresinol and (+)-pinoresinol)] and the secoiridoid derivatives [dialdehydic forms of elenolic acid linked to 3,4-DHPEA and *p*-HPEA (3,4-DHPEA-EDA and *p*-HPEA-EDA), isomer of oleuropein aglycon (3,4-DHPEA-EA) and ligstroside aglycone] were obtained as quoted in the study of Veneziani et al. (8). The analytical standards of volatile compounds [pentanal, (*E*)-2-pentenal, hexanal, (*E*)-2-hexenal, (*E, E*)-2,4-hexadienal, 2,4-hexadienal (i), 1-pentanol, 1-penten-3-ol, (*E*)-2-penten-1-ol, (*Z*)-2-penten-1-ol, 1-hexanol, (*E*)-2-hexen-1-ol, (*Z*)-3-hexen-1-ol, (*E*)-3-hexen-1-ol, hexyl acetate], α-tocopherol and all the reagents used in the analysis were purchased from Merck (Merck KGaA, Darmstadt, Germany).

### Legal Quality Parameters

The main legal quality parameters (free acidity, peroxide value, and the UV absorption characteristics) of the EVOOs were determined by the European Official Methods (30).

### Moisture Content of Pomace

The moisture contents of the control and PEF EVOOs were evaluated following the method described by International Organization for Standardization (45). Five grams of each oil sample was weighed in an aluminum capsule and placed in a BINDER oven (BINDER GmbH, Tuttlingen, Germany) at 105°C for ~5 h until a constant weight was obtained.

### Oil Content of Pomace

A Soxhlet extractor was utilized to analyze the pomace oil content; 10 g of dried sample and 5 g of pumice stone were loaded into a thimble made from thick filter paper and placed in the main compartment of the Soxhlet extractor. The process was carried out for 6 h using hexane as the extraction solvent. The solvent was removed by means of a rotary evaporator [Rotavapor R-210 (BUCHI Italia s.r.l, Cornaredo, Italy)], and the residual oil content was detected afterwards (31).

### Phenolic Compounds

**Figure 2:**
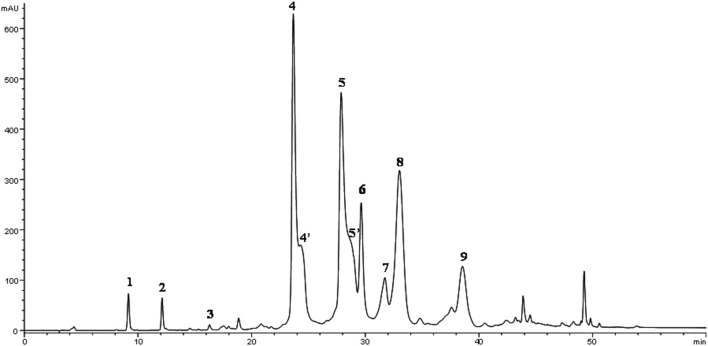
HPLC chromatogram of EVOO methanolic extract of cv. Peranzana recorded with DAD at 278 nm. Peak numbers: 1, 3,4-DHPEA; 2, p-HPEA; 3, vanillic acid; 4, 3,4-DHPEA-EDA; 5, p-HPEA-EDA; 6, (+)-1-acetoxypinoresinol; 7, (+)-pinoresinol; 8, 3,4-DHPEA-EA; 9, ligustroside aglycone [4′ and 5′ structures identified by Rovellini et al. (44)].

The phenolic fraction was recovered by a liquid-liquid extraction method mixing 20 g of EVOOs with 10 mL of methanol/water solution (80/20 v/v) using Ultra-Turrax T 25 homogenizer (IKA Labortechnik, Staufen, Germany) at 17,000 rpm for 2 min. The mixture was centrifuged at 935 × g for 10 min at room temperature (Andreas Hettich GmbH & Co.KG, Tuttlingen, Germany) than the supernatant was recovered (32). The extraction was repeated twice. The quantitative and qualitative phenolic concentrations of the EVOOs were determined by high-performance liquid chromatography (HPLC) using an Agilent Technologies model 1100 controlled by ChemStation (Agilent Technologies, Palo Alto, CA, USA). A C18 column, Spherisorb ODS-1 (250 × 4.6 mm), with a particle size of 5 μm (Phase Separation Ltd., Deeside, UK) was used. The mobile phase was composed of 0.2% acetic acid (pH 3.1) in water (solvent A) with methanol (solvent B). The gradient was changed as follows: 95% A/5% B for 2 min, 75% A/25% B in 8 min, 60% A/40% B in 10 min, 50% A/50% B in 16 min, 0% A/100% B in 14 min. This composition was maintained for 10 min and was then returned to the initial conditions and equilibration in 13 min. The final running time was 73 min with a flow rate of 1 mL min^−1^ (Figure 2). The phenolic compounds were identified and quantified according to the procedure reported by Selvaggini et al. (33).

### Volatile Compounds

The headspace, solid-phase microextraction followed by gas chromatography mass spectrometry (HS-SPME/GC-MS) technique was used to detect and quantify the volatile compounds in the control and PEF EVOOs of the Carolea, Ottobratica, and Coratina cultivars.

The SPME was carried out holding the vials, with 3 g of EVOO and 50 μL of a standard methanolic solution, at 35°C and then the SPME fiber (a 50/30 μm 1 cm long DVB/Carboxen/PDMS, Stableflex; Supelco, Inc., Bellefonte, PA, USA) was exposed to the vapor phase for 30 min to detect the volatile compounds.

**Figure 3:**
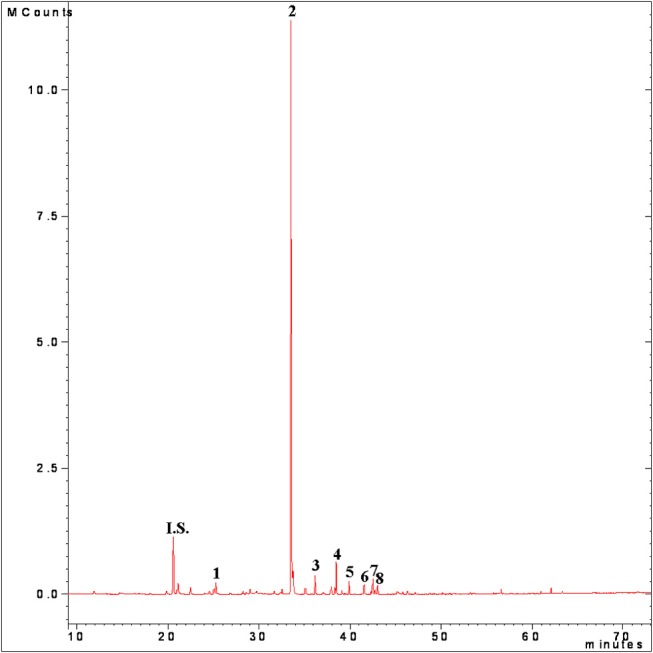
HS-SPME-GC-MS volatile fraction total ion current chromatogram of virgin olive oil cv. Peranzana. Peak numbers: I.S., internal standard (Isobutyl acetate); 1, hexanal; 2, (*E*)-2-hexenal; 3, hexyl acetate; 4, (*Z*)-3-hexenyl acetate; 5, 1-hexanol, 6, (*Z*)-3-hexen-1-ol; 7, (*E*)-2-hexen-1-ol; 8, (*E, E*)-2,4-hexadienal.

The GC-MS analysis were conducted using a Varian 4000 GC-MS equipped with a 1079 split/splitless injector (Varian). The fused-silica capillary column (DB-Wax-ETR, 50 m, 0.32 mm i.d., 1 μm film thickness; J&W Scientific, Folsom, CA, USA) was operated with helium regulated by an electronic flow controller (EFC) at a constant flow rate (1.7 ml min^−1^). All the operative conditions was set following the method described by Veneziani et al. (7) without any modifications. The data of the peak areas were evaluated on the basis of calibration curve of each different compound and expressed in μg kg^−1^ of EVVO (Figure 3).

### α-Tocopherol

The α-Tocopherol EVOOs were evaluated by HPLC–DAD–FLD analysis: 1 g of oil was dissolved in 10 mL of n-hexane, filtered with a 5-μm polyvinylidene difluoride (PVDF) syringe filter (Whatman, Clifton, NJ) and injected into the HPLC system. The HPLC analysis was conducted using the Agilent Technologies Model 1100, and the α-Tocopherol was detected at an excitation wavelength of 294 nm and at an emission wavelength of 300 nm as described by Esposto et al. (34).

### Oxidative Stability

The oxidative stability of the control and PEF EVOO of Coratina was assessed using a Rancimat (Methrom Ltd., Herisau, Switzerland) as described by Baldioli et al. (35). The oils were treated with a flow of purified air (20 L h^−1^) at 120°C for 24 h. The oxidative stability was detected as the oxidation induction time (OIT), expressed in hours.

## Results and Discussion

A PEF system was applied to the olive oil mechanical extraction process to evaluate its technological performance on the oil extractability and its impact on the main quality parameters of EVOO. Compared to the others previous studies on the effect of PEF applied to the oil mechanical extraction process, the trials were carried out by processing three different Italian olive cultivars to better understand the effects of the technology in relation to the different genetic origins of the three olive varieties. As reported by Bartolini et al. (28), Carolea, Ottobratica, and Coratina are characterized by different geographical, morphological and agronomical characters that could influence the performance of PEF system in the improvement of oil yield and EVOO quality, mainly related to phenolic and volatile compounds.

The EVOOs obtained from the PEF-assisted extraction showed an increase in the oil yield for all of the processed cultivars, indicating the efficient degradation of the olive tissues to guarantee an improvement in the oil extractability of the mechanical extraction plant. The release of a large amount of oil in the free water phase of the olive paste enhanced the total oil extracted at the end of the mechanical separation process with a variability that is a function of the different genetic origins of the olives (Table 1), with enhancement values ranging from 2.3 to 6.0%. The data were also confirmed by the analysis of oil content of pomaces that showed a lower values in the PEF samples compared to the control tests (Table 1). The above statement is in accordance with the results presented in a previous work (36), which showed that the impacts of different settings of PEF on oil extraction yields were also influenced by the cultivar and the dimension of the olive fruits. The PEF trials highlighted a putative, cultivar-dependent effect that was probably due to the different fruit-flesh/pit ratios and the moisture and oil contents of the olives, which were able to modify the power and activity levels of the electric field on the fruit cells, reducing or increasing the degradation process. Olive paste is a very complex matrix composed of different ratios of water, wood, pulp, and oil that is primarily related to the cultivar and secondarily to agronomic factors, such as the growing area, irrigation, climatic season, ripening stage, and soil management (7, 37, 38). The different elements that form the olive fruit can interfere with and influence the homogeneous diffusion of the electric field into the olive paste, altering the effects of treatment.

**Table 1:** EVOOs extraction yield, moisture and oil contents of pomaces obtained from olives Control and treated using PEF of three different cultivar^a^.

	**cv. Carolea**		**cv. Ottobratica**		**cv. Coratina**	
	**Control**	**PEF**	**Control**	**PEF**	**Control**	**PEF**
Extraction yield (%)	15.0 ± 0.3	15.9 ± 0.3	12.8 ± 0.2	13.1 ± 0.1	15.8 ± 0.4	16.6 ± 0.1
Moisture content (%)	66.8 ± 0.5	66.9 ± 0.1	59.1 ± 0.1	59.0 ± 0.2	58.7 ± 0.01	58.8 ± 0.1
Oil content (%)	7.02 ± 0.1	5.95 ± 0.2	6.01 ± 0.1	5.89 ± 0.04	5.72 ± 0.0	5.44 ± 0.1

a *The data are the mean values of four independent experiments analyzed in duplicate, ± standard deviation*.

The PEF treatment did not significantly alter the free acidity, peroxide value, or UV spectrophotometric indices of the EVOOs of any of the cultivars compared to the respective control test (Table 2).

**Table 2:** Legal quality parameters of Control and PEF EVOOs of three Italian cultivar.

	**Acidity (g of oleic acid 100 g of oil^**−1**^)**	**Peroxide value (meq of O_**2**_ kg of oil^**−1**^)**	**K_**232**_**	**K_**270**_**	**ΔK**
**cv. Carolea^a^**					
Control	0.27 ± 0.01	5.6 ± 0.1	1.668 ± 0.003	0.106 ± 0.001	−0.002 ± 0.0002
PEF	0.26 ± 0.002	6.0 ± 0.4	1.691 ± 0.01	0.111 ± 0.002	−0.002 ± 0.0001
**cv. Ottobratica**					
Control	0.28 ± 0.01	6.0 ± 0.1	1.701 ± 0.004	0.166 ± 0.005	−0.002 ± 0.0001
PEF	0.30 ± 0.01	6.2 ± 0.2	1.747 ± 0.02	0.153 ± 0.004	−0.003 ± 0.0003
**cv. Coratina**					
Control	0.27 ± 0.02	3.0 ± 0.07	1.817 ± 0.01	0.187 ± 0.02	−0.004 ± 0.0002
PEF	0.28 ± 0.01	3.1 ± 0.1	1.828 ± 0.02	0.185 ± 0.002	−0.005 ± 0.0001

a *The data are the mean values of four independent experiments analyzed in duplicate, ± standard deviation*.

**Figure 4:**
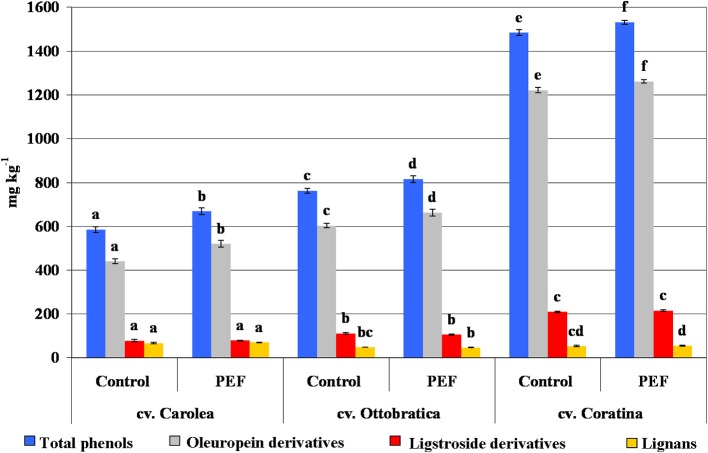
Phenolic composition (mg kg^−1^) of the PEF and control EVOOs of the three different Italian olive cultivars. Phenolic content was expressed as total phenols, oleuropein derivatives (sum of 3,4-DHPEA, 3,4-DHPEA-EDA, and 3,4-DHPEA-EA), ligstroside derivatives (*p*-HPEA, *p*-HPEA-EDA and ligstroside aglycone) and lignans [sum of (+)-1-acetoxypinoresinol and (+)-pinoresinol]. The data are the mean values of three independent extractions analyzed in duplicate, ± standard deviation. The values of each phenolic group with different letters (a–f) are significantly different from one another (*p* < 0.05).

The evaluation of hydrophilic phenols in the experimental trials showed an increase in phenolic compounds in all of the EVOOs extracted from different cultivars compared to the control test, which was confirmed by Puértolas and Martínez de Marañón (14). The overall positive effect of the PEF system on the phenolic fraction of the EVOOs led to the conclusion that the non-thermal treatment applied to the malaxed olive paste improves the release of phenols and solubilization into the oily phase. Figure 4 shows the percentage increases in the phenolic fractions of the PEF-EVOOs expressed as total phenols, oleuropein derivatives (sum of 3,4-DHPEA, 3,4-DHPEA-EDA, and 3,4-DHPEA-EA), ligstroside derivatives (*p*-HPEA, *p*-HPEA-EDA, and ligstroside aglycone) and lignans (sum of (+)-1-acetoxypinoresinol and (+)-pinoresinol). The phenolic enhancement was qualitatively due to the amount of 3,4-DHPEA-EDA and 3,4-DHPEA-EA, which are the main phenolic compounds influenced by technological processes, whereas ligstroside derivatives and lignans seemed more stable than the other molecules (4, 5, 9, 10, 39). The significant increases of total phenol, 14.3, 7.05, and 3.2% for Carolea, Ottobratica, and Coratina, respectively, showed a high variability, probably due to the different genetic origins of the olive cultivars, even if the lowest enhancement, which was detected during the extraction of the Coratina EVOOs, could be the result of saturation phenomena in the oil as a consequence of the high amount of phenols detected (~1.5 g kg^−1^), which is probably very close to the limit value of the product.

In contrast, the content of α-tocopherol was not influenced by the PEF treatment and did not show any significant differences in concentration in any of the cultivars. The concentration of lipophilic phenols was 204.2 and 203.3 mg kg^−1^ (cv. Carolea), 313.5 and 314.7 mg kg^−1^ (cv. Ottobratica), 261.3 and 266.3 mg kg^−1^ (cv. Coratina) for control, and PEF EVOOs, respectively.

**Figure 5:**
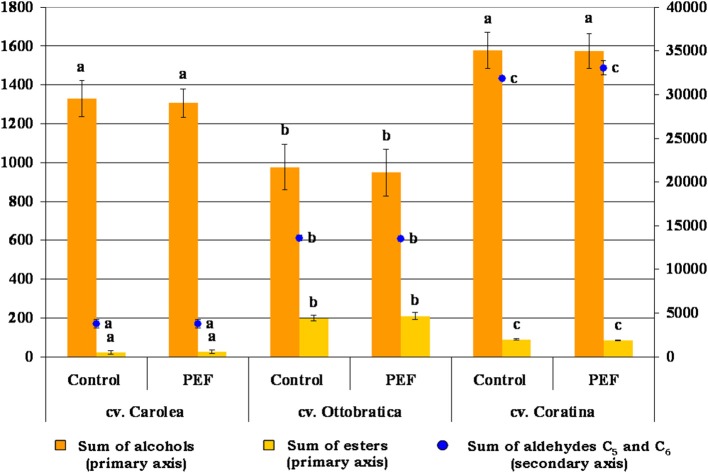
Volatile composition (μg kg^−1^) of the PEF and control EVOOs of the three different Italian olive cultivars responsible for oil flavor. Volatile content was expressed as alcohols [sum of 1-pentanol, 1-penten-3-ol, (*E*)-2-penten-1-ol, (*Z*)-2-penten-1-ol, 1-hexanol, (*E*)-2-hexen-1-ol, (*Z*)-3-hexen-1-ol, and (*E*)-3-hexen-1-ol], esters [sum of hexyl acetate and (*Z*)-3-hexenyl acetate] and C_5_ and C_6_ saturated and unsaturated aldehydes [sum of pentanal, (*E*)-2-pentenal, hexanal, (*E*)-2-hexenal, (*E,E*)-2,4-hexadienal and 2,4-hexadienal (i)]. The data are the mean values of three independent extractions analyzed in duplicate, ± standard deviation. The values of each volatile compound group with different letters (a–c) are significantly different from one another (*p* < 0.05).

The same trend was detected for the volatile fractions of the EVOOs treated using PEF technology that did not modify the concentration of the main aldehydes, alcohols, and esters involved in the flavor of the olive oils (Figure 5). The PEF system was performed in the oil extraction phase, during which the largest amount of volatile compounds were already produced and probably did not have time to interfere with the activity of the enzymes of the lipoxygenase pathway. In addition, the non-thermal treatment did not negatively alter the concentrations of the developed volatile compounds in the EVOOs, as reported by other authors in several food products (40–43).

The electrodes that generated the pulsed electric field (PEF) could release traces of metals, such as iron and cupper, characterized by pro-oxidant activities into the product, which could possibly negatively impact the oxidative stability of the food matrices. No data were found in the literature about this possible effect of PEF treatment of olive paste and its potential consequences on the EVOO quality. For that reason, the oxidative stability, analyzed by the Rancimat test, was also detected in the EVOOs extracted from the Coratina olives to evaluate the differences in the oxidation induction time (OIT). The data showed a longer OIT of the EVOO from the PEF sample (21.1 h) compared to the control (20.3 h), highlighting the absence of possible metals issued by the electrodes in the PEF oil, which should increase the rate of oil oxidation. The higher OIT for the PEF sample was due to the major concentration of antioxidant compounds (Figure 4).

## Conclusion

The impact of the PEF technology applied to the olive oil mechanical extraction process showed a significant effect on the EVOO yield for the tests conducted on Carolea, Ottobratica, and Coratina olives. The percent increase in the oil yield (ranging from 2.3 to 6%) should be cultivar dependent, probably in relation to the different ratios of the constituent parts of the fruits, which is mainly influenced by the genetic origins of the olive drupes and by the ripening stage. As also supposed in a previous study (14), the different fruit-flesh/pit ratios and the moisture and oil contents influenced PEF extraction performance. The PEF also showed a positive impact on the quality of the EVOO characterized by an enhancement of the phenolic compounds responsible for health-promoting benefits, with an increase ranging from 3.2 to 14.3% that is a function of the different olive cultivars and their maturity index.

The alteration of the olive tissue structure induced by the PEF treatment and the subsequent release of intracellular matrices into the water phase did not affect the legal quality parameters or the oxidative stability of the product as a consequence of the possible release of pro-oxidant metals from the PEF chamber. The concentrations of α-tocopherol and the main classes of volatile compounds responsible for the EVOO flavor were not significantly modified compared to the control test. The new technology improved the oil extractability and the antioxidant contents of the EVOO without altering the main qualitative and organoleptic characteristics of the product.

## Data Availability

The raw data supporting the conclusions of this manuscript will be made available by the authors, without undue reservation, to any qualified researcher.

## Author Contributions

GV: extraction process, data processing, and conclusion. SE and AT: data processing. RS and BS: chemical analysys. AL and LD: sampling and chemical analysys. MP and RT: engineering technical support. MS: research plan and supervision.

### Conflict of Interest Statement

The authors declare that the research was conducted in the absence of any commercial or financial relationships that could be construed as a potential conflict of interest.
